# Pulmonary Valve Morphology in Patients with Bicuspid Aortic Valves

**DOI:** 10.1007/s00246-018-1807-x

**Published:** 2018-01-16

**Authors:** Wilke M. C. Koenraadt, Margot M. Bartelings, Adriana C. Gittenberger-de Groot, Regina Bökenkamp, Marco C. DeRuiter, Martin J. Schalij, Monique R. M. Jongbloed

**Affiliations:** 1 Department of Cardiology, Leiden University Medical Center, P.O. Box 9600, 2300 RC Leiden, The Netherlands; 2 Department of Anatomy & Embryology, Leiden University Medical Center, Leiden, The Netherlands; 3 Department of Paediatric Cardiology, Leiden University Medical Center, Leiden, The Netherlands

**Keywords:** Bicuspid aortic valve, Pulmonary valve, Morphology, Congenital heart disease, Valvular disease

## Abstract

The aortic and pulmonary valve share a common developmental origin from the embryonic arterial trunk. Bicuspid aortic valve is the most common congenital anomaly and can occur isolated as well as in association with other congenital heart disease (CHD). Data on pulmonary valve morphology in these cases are scarce. In this study, we aimed to determine pulmonary valve morphology in hearts with BAV associated with CHD. In 83 post-mortem heart specimens with BAV and associated CHD, pulmonary valve morphology was studied and related to BAV morphology. In 14/83 (17%) hearts, the pulmonary valve was affected, bicuspid in 8/83 (10%), dome-shaped in 3/83 (4%) and atretic in 3/83 (4%). In specimens with a bicuspid pulmonary valve, 5/8 (63%) had a strictly bicuspid aortic valve (without raphe), 2/3 hearts (67%) with dome-shaped pulmonary valves and 2/3 hearts (67%) with atretic pulmonary valves had BAV without raphe. Six out of eight (75%) specimens with a bicuspid pulmonary valve had a perimembranous ventricular septal defect (VSD). 4/8 (50%) specimens with a bicuspid pulmonary valve were associated with chromosomal abnormalities: 3 (38%) had trisomy 18 and 1 (13%) had trisomy 13. In BAV with associated CHD, abnormal pulmonary valve morphology was observed in 17% of the hearts. The majority of hearts with abnormal pulmonary valve morphology had a Type B bicuspid aortic valve (without raphe). Bilateral semilunar valvular disease is associated with Type B BAVs and in many cases related to chromosomal abnormalities. As this study was performed in post-mortem specimens with high frequency of associated CHD, caution is warranted with application of these results to the general BAV population.

## Introduction

Bicuspid aortic valve (BAV) is the most common congenital anomaly, occurring in 1–2% of the general population [1], whereas bicuspid pulmonary valves are rare (incidence of 0.1%) [2]. The aortic valve and the pulmonary valve arise from a common arterial trunk, which is initially unseparated. During development, separation at the level of the outflow tract, valves and great vessels needs to occur. Neural crest cells initiate the aorto-pulmonary septum formation, after which a separate aorta and pulmonary valve can be distinguished [3].

Bicuspid pulmonary valves are usually associated with other congenital heart diseases such as tetralogy of Fallot [4] or transposition of the great arteries [5]. Bilateral bicuspid semilunar valves are rare and diagnosed mainly during surgery [6] or post-mortem [2]. One study reports a case of bicuspid pulmonary and aortic valves diagnosed by three-dimensional transesophageal echocardiography [7]. Animal studies report a higher incidence of bilateral bicuspid semilunar valves, up to 4% in the Syrian hamster [8]. One study in human post-mortem hearts reports an incidence of 12%, mostly in hearts with associated congenital heart disease (CHD) [9]. Given the common developmental origin of aorta and pulmonary valves [10], the question arises whether pulmonary valve morphology is affected in specific subgroups of BAV patients. In general, this does not necessarily seem the case. Animal studies have pointed out that bicuspid aortic and pulmonary valves may have a different morphogenetic origin, at least in valves not associated with major malformations of the heart [8]. Studies in humans, especially in hearts with associated CHD, are however scarce [2, 6, 9] and the relation with BAV morphology (i.e. location and presence of raphe) has not been studied to date.

The aim of the current study is to describe the incidence of abnormal pulmonary valve morphology in post-mortem hearts with BAV and associated congenital heart disease (CHD), and to correlate findings with BAV morphology.

## Methods

### Study Population

This study was performed in accordance with the local ethics committee and Dutch regulations for proper use of human tissue for medical research purposes. Eighty-four post-mortem heart–lung specimens with BAV from the Leiden collection of malformed hearts (Department of Anatomy & Embryology, Leiden University Medical Center, Leiden, The Netherlands) were studied macroscopically. These hearts were obtained from autopsies and preserved in an ethanol/glycerin solution, collected from the early 1950s until now. Two experienced observers investigated the hearts. Cardiac morphology was assessed using sequential segmental analysis [11]. All cardiovascular anomalies as well as detailed morphology of the aortic valve and pulmonary valve were noted. In some cases, cardiovascular anomalies could not be assessed due to incompleteness of the specimens. Coronary anatomy in these hearts was described in a separate paper [12].

### Valve Classification and Terminology

**Fig. 1:**
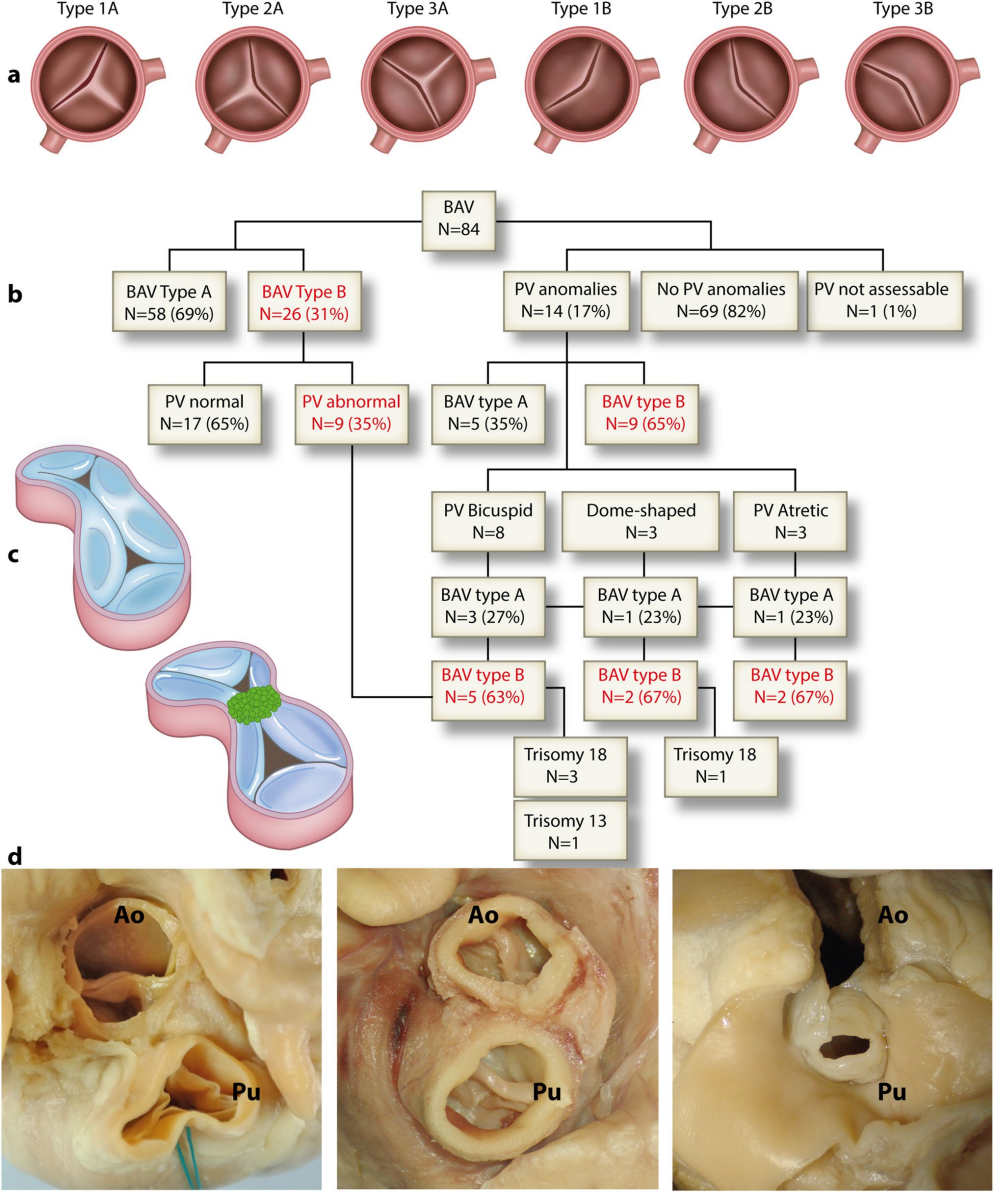
**a** Schematic overview of different BAV morphologies (Modified after [13]). **b** Schematic overview of results in the study population. **c** Development of semilunar valves. The initially unseparated common arterial trunk contains endocardial cushions (blue), that will be separated, orchestrated by neural crest cells (green dots). **d** Examples of post-mortem heart specimens with variable pulmonary valve morphology. *Left* Type 1A BAV and tricuspid pulmonary valve. *Middle* Type 3B BAV and bicuspid pulmonary valve. No raphe can be determined in the pulmonary valve. *Right* Type 1B bicuspid aortic valve and dome-shaped pulmonary valve

BAV morphology was defined by orientation of leaflets with respect to each other, based on attachment of leaflets (right coronary, left coronary, non-coronary) and the presence and position of a raphe. BAV morphology was classified as *Type 1* if right and left coronary leaflets were conjoined; *Type 2* if right and non-coronary leaflets were conjoined and *Type 3* if left and non-coronary leaflet were conjoined. ‘A’ was added for valves in which a raphe could be recognized or ‘B’ for strictly bicuspid valves (no raphe) (Fig. 1a). A raphe was defined as a ridge located in the conjoined area of two leaflets, presumably representing a malformed commissure. Subsequently, pulmonary valve morphology (tricuspid, bicuspid, unicommissural/dome-shaped or atretic) was assessed as well as the presence or absence of a raphe.

## Results

The study group consisted of 84 specimens, 1 specimen was excluded due to non-assessable pulmonary valve morphology. Age at demise ranged from foetal to 24 years. Of the 83 patients, 24 died of cardiac causes, for example, heart failure or arrhythmia. Twenty-five patients died perioperative or immediately postoperative. Twenty-four patients died of non-cardiac causes, such as chromosomal abnormalities, intracerebral bleeding or pulmonary problems. For ten patients, cause of death was unknown.

Characteristics of the specimens are listed in Table 1.

**Table 1:** Patient characteristics and associated cardiovascular abnormalities

Complete study group	*n* ^a^	%
Gender (male:female)	48:35	
**Aortic valve morphology**		
Type 1A	45/84	54
Type 2A	9/84	11
Type 3A	5/84	6
Type 1B	16/84	19
Type 2B	5/84	6
Type 3B	4/84	5
**Pulmonary valve morphology**		
Tricuspid	69/83	83
Dome-shaped	3/83	4
Bicuspid	8/83	10
Atresia	3/83	4
**Associated anomalies**		
Aortic arch anomalies	46/77	60
*Hypoplasia*	13/77	17
*Coarctation*	33/77	43
ASD	11/83	13
AVSD	4/84	5
AV valve anomalies		
*TV anomalies*	15/68	22
*MV anomalies*	30/50	60
DORV	6/83	7
PLSCV	15/83	18
TGA	2/74	3
VSD	42/84	50
*Muscular*	20/84	24
*Perimembranous*	22/84	26
**Chromosomal abnormalities**		
Trisomy 9	1	
Trisomy 13	2	
Trisomy 18	5	

*ASD* atrial septal defect; *AVSD* atrioventricular septal defect; *AV valve* atrioventricular valve; *TV* tricuspid valve; *MV* mitral valve ; *DORV* double outlet right ventricle; *PLSCV* persistent left superior caval vein; *TGA* transposition of the great arteries; *VSD* ventricular septal defect; *PM VSD* perimembranous VSD

^a^The number of observed cases that could be assessed for this particular abnormality

In 69/83 (83%) specimens (42 male, 26 female, 1 unknown) the pulmonary valve was normal (Fig. 1b). The majority of the 83 included specimens had associated congenital anomalies (Table 1). In 14/83 (17%) hearts, the pulmonary valve was affected; the pulmonary valve was bicuspid in 8/83 hearts (10%), dome-shaped in 3/83 hearts (4%) and three pulmonary valves were atretic (4%). Examples are shown in Fig. 1d.

Of the 14 specimens with an affected pulmonary valve, 9 (64%) had a Type B (strictly bicuspid) aortic valve (Fig. 1b; Table 2). For comparison, in the complete study group, 26/84 hearts (31%) had Type B BAV.

**Table 2:** Associated cardiovascular abnormalities in patients with pulmonary valve anomalies

Subgroup with pulmonary valve anomalies (*n* = 14)	AoV morphology	Associated anomalies
Dome-shaped (*n* = 3)	1A	ASD, TV dysplasia
	1B	Muscular VSD
	3B	PLSCV, PM VSD, trisomy 18
Bicuspid (*n* = 8)	1A	DORV, PM VSD
	1B	TV hypoplasia, arch hypoplasia, PM VSD, trisomy 18
	2A (*n* = 2)	PLSCV, ASD, TV dysplasia, PM VSD; MV hypoplasia, arch hypoplasia, PM VSD
	2B	PLSVC, PM VSD, trisomy 18
	3B (*n* = 3)	Trisomy 13; PLSVC, TV dysplasia; PLSCV, PM VSD, trisomy 18
Atretic (*n* = 3)	1A	AVSD, DORV
	1B	DORV, TV dysplasia, MV hypoplasia, PM VSD
	3B	Muscular VSD

*ASD* atrial septal defect; *AVSD* atrioventricular septal defect; *AV valve* atrioventricular valve; *TV* tricuspid valve; *MV* mitral valve; *DORV* double outlet right ventricle; *PLSCV* persistent left superior caval vein; *TGA* transposition of the great arteries; *VSD* ventricular septal defect; *PM VSD* perimembranous VSD

In the subgroup of specimens with a bicuspid pulmonary valve, 5/8 (63%) also had a strictly bicuspid aortic valve (1 Type 1B, 1 Type 2B and 3 Type 3B, Fig. 1b; Table 2). Of these, 1 pulmonary valve was also strictly bicuspid, the other 4 did have a raphe. In 3/8 (38%) hearts with a bicuspid pulmonary valve, a raphe was present (Type A): 1 of these was Type 1A BAV and 2 were Type 2A (Table 2).

Of the 3 hearts with dome-shaped pulmonary valves, 2 (67%) had a Type B BAV (1 Type 1B and 1 Type 3B BAV, Fig. 1b; Table 2), whereas 2/3 hearts (67%) with atretic pulmonary valves had a Type B BAV (1 Type 1B and 1 Type 3B).

In 5/14 specimens (36%), not only the semilunar valves but also the atrioventricular (AV) valves were affected (Table 2).

Of the 8 specimens with a bicuspid pulmonary valve, 6 (75%) had a perimembranous ventricular septal defect (VSD).

4/8 (50%) specimens with a bicuspid pulmonary valve were associated with chromosomal abnormalities: 3 (38%) had trisomy 18, 1 (13%) had trisomy 13 and these all had a Type B BAV.

## Discussion

Key findings of the current study are as follows: (1) In the BAV population with associated CHD, abnormal PV morphology was encountered in 17% of hearts. (2) The majority of hearts with abnormal pulmonary valve morphology had a Type B BAV. (3) Bilateral bicuspid semilunar valves were associated with chromosomal abnormalities in 50% of specimen, predominantly trisomy 18.

Given the common developmental background of the aortic and pulmonary valves both derived from outflow tract cushions with contributions of endocardium-derived cells, neural crest cells [10] and, possibly, epicardial-derived cells [14], one could expect that in case of BAV also the pulmonary valve is affected. The observed 17% of the cases (bicuspid, dome-shaped and atretic pulmonary valves) in the current study, is a minority but still a higher percentage than reported in previous studies [2, 6], indicating at least a partly common development of aorta and pulmonary valves. Almost two-third of the hearts with an abnormal pulmonary valve had a strictly bicuspid aortic valve, indicating a potential predisposition of maldevelopment of the pulmonary valve in a subgroup of patients with BAV Type B. Although the mechanism is unclear at this point, it seems that in cases where tripartition of the valve is severely disturbed to the extent that an anatomical third leaflet and a raphe cannot be recognized, the development of the pulmonary valve (that evolves from the same endocardial cushions that contribute to the aorta during development, Fig. 1c) is also prone to be affected. In this respect, it is interesting that in 75% of the hearts with bilateral semilunar valves, there was a perimembranous VSD, suggesting an early defect in endocardial cushion remodelling, as these cushions are also involved in closure of the interventricular foramen.

In 10% of the specimens, there was concurrence of a bicuspid aortic and pulmonary valve, which is a similar percentage as has been found in a previous study [9]. The association with chromosomal abnormalities, especially a higher incidence of Trisomy 18, is in accordance with previous studies [9, 15, 16].

The fact that not only the semilunar valves but also the AV valves were affected in 36% of the cases, as well as the high percentage of perimembranous VSDs that were observed, could indicate a developmental cause affecting primarily endocardial cushion-derived tissues [17].

## Conclusion

Pulmonary valve pathology was encountered in 17% of the cases with BAV. Two-third of these cases had a BAV Type B. In addition, over one-third of specimens with abnormal pulmonary valve morphology had a chromosomal abnormality, and all of these had a BAV Type B. We conclude that bilateral semilunar valve disease is associated with Type B BAVs, in many cases related to chromosomal abnormalities. As this study was performed in post-mortem specimens with high frequency of associated CHD, caution is warranted with application of these results to the general BAV population.

## Study Limitations

This study was performed in post-mortem specimens with high frequency of associated CHD and therefore cannot be applied to the general BAV population.
